# Honey-Based Templates in Wound Healing and Tissue Engineering

**DOI:** 10.3390/bioengineering5020046

**Published:** 2018-06-14

**Authors:** Benjamin A. Minden-Birkenmaier, Gary L. Bowlin

**Affiliations:** Department of Biomedical Engineering, University of Memphis, 3806 Norriswood Ave., Memphis, TN 38152, USA; bmndnbrk@memphis.edu

**Keywords:** tissue engineering, tissue regeneration, electrospinning, cryogel, hydrogel, Manuka honey, chronic wound, Inflammation

## Abstract

Over the past few decades, there has been a resurgence in the clinical use of honey as a topical wound treatment. A plethora of in vitro and in vivo evidence supports this resurgence, demonstrating that honey debrides wounds, kills bacteria, penetrates biofilm, lowers wound pH, reduces chronic inflammation, and promotes fibroblast infiltration, among other beneficial qualities. Given these results, it is clear that honey has a potential role in the field of tissue engineering and regeneration. Researchers have incorporated honey into tissue engineering templates, including electrospun meshes, cryogels, and hydrogels, with varying degrees of success. This review details the current state of the field, including challenges which have yet to be overcome, and makes recommendations for the direction of future research in order to develop effective tissue regeneration therapies.

## 1. Introduction

Honey has been used as a wound treatment by indigenous cultures around the globe for thousands of years. Archeological findings and early written works indicate that wounds were treated with honey by the ancient Egyptians, Greeks, and Romans, among others [1]. With the advent of antibiotics in the 1940s, honey fell out of favor as a wound treatment [2]. However, with the increasing prevalence of antibiotic-resistant bacteria, as well as new in vitro and in vivo data supporting honey’s effectiveness in treating wounds and as a natural broad-band antibacterial agent, it has recently made a comeback in clinical medicine. Additionally, honey’s ability to aid in situ cellularization and regeneration of implanted acellular tissue-engineered structures indicates its potential as a tissue engineering additive.

Honey is a natural substance produced by a variety of honeybee species around the world. First, the bees collect nectar from flowering foliage. This nectar is processed in an internal pouch called the crop, where a variety of enzymes break down sugars. The resulting solution is regurgitated by the bees into honeycomb within their hives, where liquid evaporation is enhanced by air currents created by the fanning of bee wings. The product is a highly concentrated viscous solution of floral sugars and proteins, enzymes, and amino acids derived from the bee crops [3]. These sugars are primarily fructose and glucose, with smaller amounts of maltose, sucrose, and isomaltose, and comprise approximately 80% of honey components, with water comprising <18% [4,5,6]. Glucose oxidase from the bee crop slowly breaks down glucose into gluconic acid, which lowers the pH of honey, and hydrogen peroxide, which helps kill bacteria [7]. In a wound site, the lower pH of honey (3.5–4) reduces protease activity, increases oxygen release from hemoglobin, and stimulates the activity of macrophages and fibroblasts, while the hydrogen peroxide content sterilizes the wound and stimulates vascular endothelial growth factor (VEGF) production [7]. Invertase, another enzyme from the bee crop, slowly divides sucrose into glucose and fructose, increasing the strength of the osmotic potential. In addition, flavenoids derived from the floral nectar sources neutralize free radicals created by the hydrogen peroxide [7]. Bees can also make honey from honeydews, a loose term which includes plant secretions and plant-sucking insect excretions [8]. These honeydew honeys have lower glucose and fructose content and higher levels of oligosaccharides [9]. Some research has also shown that honeydew honeys contain higher levels of phenolic contents, which have been shown to reduce MMP-9 expression in keratinocytes [10,11]. These findings indicate that honeydew honey may be a beneficial future focus of wound healing and tissue engineering research. However, as most honey used in tissue engineering research thus far originates with floral nectar, this review will focus on these nectar-based honeys.

Although some honey varieties have been shown to have beneficial effects in a wound site, most modern research has focused on a particular variety produced in New Zealand from the nectar of the *Leptospermum Scopartum* shrub, called Manuka honey. This honey contains the components of other honey varieties, but its unique component, methylglyoxal, acts as an additional antibacterial agent [12,13,14,15]. Several companies collect, pool, filter, and sterilize Manuka honey for clinical use, including ManukaGuard (located in New Zealand) and Medihoney (a subsidiary of Derma Sciences, Princeton, NJ, based in the United States). This collection and pooling of the honey helps limit batch-to-batch variability between hive locations and times of the year, while the filtration removes wax, dirt, and pollen particulates from the honey to reduce the potential to cause an allergic reaction. Although honey has been demonstrated to have antibacterial properties, these products are still sterilized via gamma irradiation or pasteurization to doubly ensure that no live bacteria or spores are present.

Honey performs several other functions as a wound covering. As a viscous fluid, its thick consistency forms a barrier between the wound and the external environment, protecting against bacteria and keeping the wound hydrated [16]. Its high concentration of sugars and other solutes creates a strong osmotic gradient that pulls fluid up through the subdermal tissue [17]. The water activity of honey, a measure of its osmotic potential, has been reported to range from 0.53 to 0.64 aw (activity of water, unitless) [18,19]. For reference, the water activity of distilled water is 1 aw, and substances with a lower water activity create a higher osmotic potential with water flowing from areas of high to low water activity. Water activity values below 0.91 aw inhibit bacterial growth [20]. The low water activity of honey causes fluid flow which flushes bacteria, debris, slough, and necrotic tissue out of the wound, and carries nutrients and oxygen from the deep tissue into the wound area. Additionally, the low pH of the honey increases tissue oxygenation, while the flavonoids and aromatic acids scavenge free radicals, preventing tissue damage and controlling inflammation [7,21]. The high sugar content of honey also provides an additional source of glucose for proliferating cellular components (i.e., fibroblasts and endothelial cells) in the area [16].

In addition to these other attributes, honey also has multiple antibacterial effects. These effects include the inhibition of bacterial growth as well as the direct killing of bacteria [22,23,24,25]. The osmotic potential of the honey crenates bacteria at the top of the wound, destroying them [15,26]. Although this osmotic potential was thought by some groups to be the main source of honey’s antibacterial activity, studies have shown that honey maintains its antibacterial activity even when diluted by wound exudate [27,28]. An in vitro study by Cooper et al. found that dilutions of honey by factors of 7 to 14 maintain their bacterial inhibition, long past the dilution point where the osmotic potential of the solution would cease to be bactericidal [29]. It should be noted that this study lacked mechanistic controls of concentrated sugar solutions with equal water activity to honey, weakening their results. This lack of mechanistic controls is a general issue for some studies of honey’s antibacterial effects, making it difficult to measure the contribution of each attribute to the overall antibacterial nature of honey. Nevertheless, honey has been shown to contain other components which contribute to its bactericidal effect. As mentioned above, honey contains hydrogen peroxide, with levels in the range of 12–72 µg/mL depending on the dilution of the honey and variety of the honey (it should be noted that some varieties of honey do not contain measurable levels of hydrogen peroxide, discussed further below) [22]. Hydrogen peroxide easily gives up one of its oxygen atoms to the surrounding environment, creating a free radical that causes oxidative damage to bacterial cell walls. Additionally, the presence of bee defensin-1 has been shown in some honey varieties, although the levels of this protein vary based on hive location [30,31]. Like other defensins, bee defensin-1 permeabilizes bacteria and inhibits their RNA, DNA, and protein synthesis [32]. As mentioned above, the glucose oxidase content of honey lowers its pH, which can also kill some bacteria [30]. Manuka honey, in particular, contains methylglyoxal, a compound which has been shown to damage bacteria flagella and thus limit their mobility and ability to adhere to surfaces [33]. However, Manuka honey has been shown to lack the defensin-1 content of other honey varieties, possibly due to decreased secretion by bees during the formation process [30,34]. The methylglyoxal content of Manuka honey has also been shown to inactivate defensin-1 when it is added to the honey, eliminating its contribution to the antibacterial effect [34]. Additionally, methylglyoxal crosslinks glucose oxidase, destroying its enzymatic activity and eliminating the hydrogen peroxide content in Manuka honey [35]. These studies utilized mechanistic controls of methyglyoxal alone or added to non-methylglyoxal-containing honeys to isolate the effect of the methylglyoxal on the other honey components. In at least one other study, however, the methylglyoxal component of honey has been neutralized to determine whether it is the sole contributor to the antibacterial effects of Manuka honey. Although this methylglyoxal-neutralized honey had decreased activity against *Staphylococcus aureus* and *Bacillus subtilis*, it did not have reduced activity against *Escherichia coli* or *Pseudomonas aeruginosa.* As such, other components besides methylgyoxal must contribute to the antibacterial activity of Manuka honey [22]. Manuka honey has been shown to be especially useful against antibiotic-resistant bacteria [12,36]. The many functions of Manuka honey thus not only clear wound debris, maintain hydration, control inflammation, and stimulate healing, but also sterilize the wound. Although a large number of groups researching honey as a wound treatment or in tissue-engineered templates have focused on Manuka honey, there are some notable studies which have examined other honey varieties and found them to have beneficial effects comparable to those of Manuka honey. Accordingly, while this paper includes a great deal of information about Manuka honey, its scope has been widened to include pertinent research into other varieties of honey.

As the focus of this review is on the use of honey in tissue engineering templates, it is not a comprehensive discussion of the components of honey, their contribution to its wound-healing mechanisms, or the entire body of research into honey’s effects as a wound additive. Rather, these topics are summarized as they relate specifically to the incorporation of honey into tissue engineering templates. For more in-depth research into the components of honey, the reader should seek out “The components of honey and their effect on its properties: a review” by Thawley or “Major components of honey analysis by near-infrared transflectance spectroscopy” by Garcia-Alvarez et al. [37,38]. For a review of the anti-bacterial mechanisms of honey and quantitative studies of their contributions to the overall anti-bacterial nature of honey, readers should obtain “How honey kills bacteria” by Kwakman et al. or “Antibacterial components of honey” by Kwakman and Zaat [6,30]. If a more comprehensive review of the mechanisms of honey that contribute to wound healing is desired, the authors suggest “Honey: a potent agent for wound healing?” by Lusby et al. or “The evidence and the rationale for the use of honey as a wound dressing” by Molan [39,40].

## 2. In Vitro and In Vivo Evidence of the Beneficial Effects of Honey in Wounds

### 2.1. Antibacterial and Antibiofilm Effects of Honey

Studies have examined the anti-bacterial action of Manuka honey against a variety of pathogens. Sherlock et al. used agar plate well diffusion assays and a spectrophotometric minimum inhibitory concentration assay to demonstrate antibacterial effects. These effects were quantified for both Manuka honey and Ulmo honey, a strain from Chile. The results of these experiments, shown in Table 1, demonstrated that both Manuka and Ulmo honey significantly inhibited the growth of *E. coli*, *P. aeruginosa*, and methicillin-resistant S. *aureus* (MRSA). Interestingly, the Ulmo honey was more effective against MRSA, although slightly less effective against *E. coli* and *P. aeruginosa* [41]. Jenkins et al. also reported that Manuka honey inhibits the growth of MRSA, and showed that the presence of honey causes a downregulation of universal stress protein A (UspA) in the MRSA, reducing its stress stamina response [12]. In addition to its effectiveness against MRSA, Cooper et al. showed that Manuka honey also inhibits the growth of at least seven different strains of vancomycin-resistant enterococci [15]. Manuka honey also has been shown to be effective against *Helicobacter pylori*, the cause of most stomach ulcers [42]. Research by Watanabe et al. in 2014 showed that Manuka honey inhibits influenza viral replication, enhancing the effects of antiviral drugs [43]. This work has been replicated with varicella and rubella viruses, indicating an exciting new avenue for the clinical use of Manuka honey [44,45].

Investigators have also examined the ability of various honey types to inhibit biofilm formation or kill biofilm-embedded bacteria. Marckoll et al. tested the effects of Manuka honey and Norwegian Forest honey on biofilm-embedded MRSA, methicillin-resistant *S. epidermidis* (MRSE), extended-spectrum β-lactamase (ESBL) *Klebsiella pneumoniae*, and *P. aeruginosa*. This study found that the active components of the honey diffused through established biofilm matrices of all bacterial types and killed bacteria in a dose-dependent manner, with minimum concentrations between 6 and 12% of Manuka honey and 12 to 25% of Norwegian Forest honey killing biofilm-embedded bacteria depending on the bacterial type. While the presence of a biofilm did provide some protection to the MRSA, MRSE, and ESBL *Klebsiella*, no protection was observed in the *P. aeruginosa* biofilm [46]. Similarly, Bardy et al. tested the ability of Manuka honey and an Australian non-methylglyoxal-containing honey, Capilano honey, to inhibit biofilm formation of *S. aureus* strains isolated from clinical patients. Biofilm inhibition was found to be related to methyglyoxal content, with a minimum level of 0.53 mg/mL methyglyoxal in Manuka honey solution necessary for biofilm-cidal activity (a 33% *w*/*v* level of Manuka honey). By itself, the Capilano honey solutions did not inhibit biofilm formation, but when at least 1.05 mg/mL methylglyoxal was added they were able to become biofilm-cidal [47]. The difference in minimum methylglyoxal levels necessary to inhibit biofilm formation between these two honey types indicates that while methylglyoxal content is important, there is an additional unknown biofilm-cidal component present in Manuka honey that is not present in the Capilano variety.

Likewise, Alandejani et al. demonstrated the effectiveness of Manuka honey and Sidr honey, another methylglyoxal-containing variety, against biofilms of MRSA, methicillin-susceptible *S. aureus* (MSSA), and *P. aeruginosa*. Both honeys were tested at a 1:2 dilution level and found to inhibit the growth of most samples of each bacterial strain. However, no attempt was made to test further dilutions of these honeys or any non-methylglyoxal-containing honey variety [48]. In another attempt, Okhiria et al. tested concentrations of 0%, 20%, and 40% *w*/*v* Manuka honey on biofilms formed by six cultures of *P. aeruginosa* and found that biofilm shrinking only occurred at the 40% *w*/*v* level [49]. A more thorough study by Sojka et al. utilized a multispecies wound biofilm model containing *S. aureus*, *Streptococcus agalactiae*, *Enterococcus faecalis*, *P. aeruginosa* and *K. pneumoniae* to test non-diluted Manuka honey, Honeydew honey, and an artificial honey prepared from fructose, glucose, maltose, and sucrose (mechanistic control). While the artificial honey was somewhat effective against *P. aeruginosa*, decreasing the number of colony-forming units from around 10^6^ c.f.u./mg to around 10^4^ c.f.u./mg over a period of 48 h, it did not significantly kill or inhibit the other three bacterial strains. In contrast, both honey varieties significantly decreased *S. aureus* growth from around 10^6^ c.f.u./mg to around 10^3^ c.f.u./mg and decreased *S. agalctiae* and *P. aeruginosa* growth to around 0 c.f.u./mg over a 48-h period. This difference in bactericidal activity between the honey varieties and artificial honey indicates that while the osmotic pressure of the honeys plays a role in some anti-biofilm activity, it does not account for all of this activity in the honey. None of the natural or artificial honey types had an effect on *E. faecalis* growth, indicating that this bacterial strain was not susceptible to the antibacterial effects of the honey. The resilience of this strain to honey should be noted for future clinical application [50].

There is also in vivo evidence of the antibacterial effects of honey in wounds. A 2010 study by Moghazy et al. followed the treatment of 30 diabetic foot ulcers with commercial honey over a three-month period. A number of microorganisms were isolated from the ulcers at the beginning of the study, including *Staphylococcus aureus*, *E. coli*, *Proteus*, *Klebsiella*, and *Providencia.* All of these microorganisms were eradicated by the end of the three-month study. *Staphylococcus epidermidis,* a benign pathogen commonly found on human skin and thought to provide a reservoir of resistance genes to other infections, was isolated from 28 of the patients at the end of the study [51,52]. The presence of healthy *S. epidermidis* is a sign of healing in these wounds. While the results of this study are encouraging, it would have benefited from a non-honey treatment control group to establish an effective comparison to the current gold-standard treatments. In another study, Efem et al. used topical commercial honey treatment to treat 59 cases of non-healing ulcers. Swabs from 51 of the wounds before treatment indicated the presence of *P. pyocyanea*, *E. coli*, *S. aureus*, *Proteus mirabilis*, *Klebsiella*, *S. faecalis*, and *Streptococcus pyogenes*, while swabs performed after one week of honey treatment indicated the eradication of these microorganisms [53]. In addition to treatments with commercial store-bought honey, some studies have focused specifically on treatment with medical-grade Manuka honey. Gethin et al. compared Manuka honey treatment with a commercially available hydrogel dressing in 108 patients with sloughy infected venous leg ulcers. MRSA was identified in 16 of the wounds, 10 of which were treated with the honey while six were treated with the commercially available hydrogel. After four weeks of treatment, MRSA was eradicated in seven of the ten honey-treated wounds but only one of the six hydrogel-treated wounds [54]. These studies provide evidence supporting the use of Manuka honey as an anti-bacterial wound sterilizing agent.

As honey is cheaper than many antibiotics and has not yet been shown to induce resistant bacteria, it is likely to become a useful alternative to antibiotics in the field of wound care. However, it should be noted that biofilms of certain bacteria, such as *E. faecalis*, are resistant to the antibacterial effects of honey, which could complicate its use in clinical practice. Additionally, the literature concerning honey’s efficacy against biofilms suggests that high concentrations (at least 33% *w*/*v*) of honey are necessary. This is complicated by cytotoxicity concerns discussed later in this review. Thus, care will have to be taken to tailor the amount and dilution of honey to specific situations in clinical practice. Larger, undiluted amounts of honey may be appropriate when fighting persistent infection, while smaller, more dilute amounts of honey are likely optimal when treating inflammation and inducing tissue infiltration and regeneration.

An additional benefit of using honey in therapeutic products is that these natural antibacterial properties give it an extremely long shelf life. In sealed containers, honey remains stable for hundreds or even thousands of years, and it is often used to increase the shelf life of other food products [55,56,57,58,59]. Many antibiotics have limited shelf lives even under refrigeration—for example, penicillin in solution has a shelf life of twelve months at 10 °C [60]. In contrast, honey’s robust thermal stability allows it to go un-refrigerated and still maintain its properties indefinitely [61]. This is a major advantage, as it eliminates the need for a “cold chain” of constant refrigeration and, therefore, reduces costs substantially. The elimination of the cold chain is a particular benefit in rural areas or developing countries where there is less access to refrigeration and power interruptions can be frequent [62,63].

### 2.2. Immunomodulatory Effects of Honey

**Figure 1:**
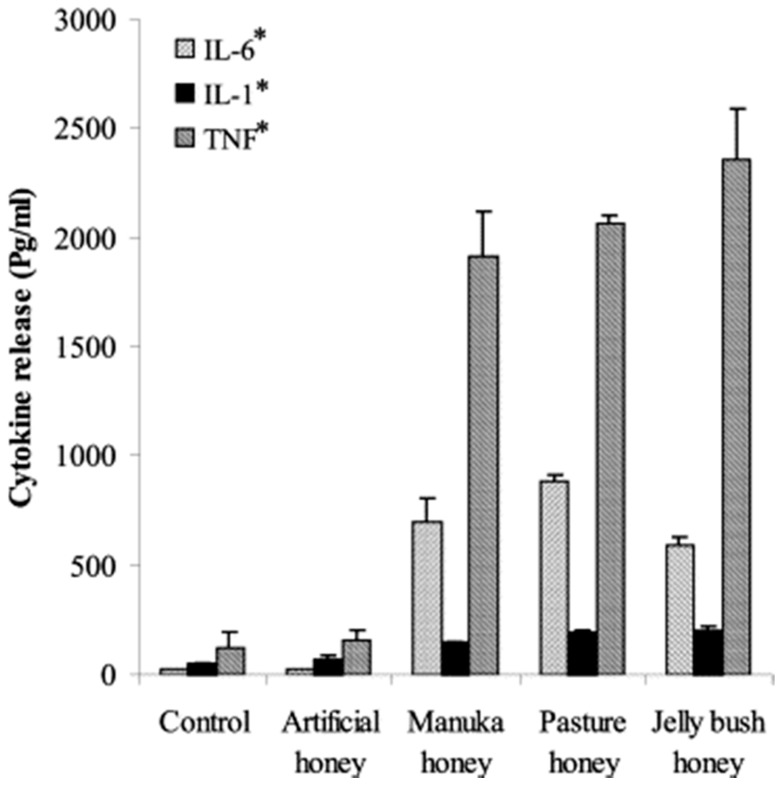
Honeys induce monocyte inflammation response. IL-6, IL-1, and TNF release from peripheral blood monocytes over 18 h in the presence of artificial honey (syrup control), Manuka honey, Pasture honey, and Jelly Bush honey. “*” indicates statistical significance (*p* < 0.001, analyzed by ANOVA with a Tukey pair-wise comparison). Reproduced with permission from Tonks et al., *Cytokine*; published by Elsevier, 2003.

A number of studies have examined the effects of honey on the immune response, with results that paint an intriguing but incomplete picture. Tonks et al. tested the monocyte response to several honey types, including Manuka honey, and observed that all honey types tested caused an increase in the release of the inflammatory mediators tissue necrosis factor α (TNF-α), interleukin 6 (IL-6), and interleukin 1 (IL-1) over a 24-h culture period, as shown in Figure 1 [64]. Of the three honey varieties tested (all 1% *v*/*v* in culture medium), Manuka honey caused the lowest release of these three inflammatory cytokines, but these levels were still significantly higher than in the non-honey controls. Specifically, Manuka honey caused an increase of about 2000 pg/mL in TNF-α release, about 100 pg/mL in IL-1 release, and about 700 pg/mL in IL-6 release over 18 h of culture as measured relative to non-honey controls. This finding would seem to indicate that these honey varieties elicit an inflammatory reaction, in direct contrast to the studies discussed below.

In another study, Leong et al. examined the effect of 21 New Zealand honey types, including varieties of Manuka honey, on neutrophil superoxide production. Their results indicate that all honey types tested reduced superoxide production in a dose-dependent manner, and this decrease in superoxide production was independent of the methylglyoxal content of the honey samples. Cytotoxicity testing revealed that at the 50% inhibitory concentrations (IC_50,_ ranging from 3.1 mg/mL to 44.4 mg/mL depending on the honey variety) of honey on superoxide production, none of the honey varieties caused significant amounts of neutrophil death [13]. However, honey treatments in wounds typically involve direct application of honey to the wound at much higher concentrations than these IC_50_s, throwing doubt into the relevance of this finding to wound treatment. This study also involved an in vivo murine test which measured the effect of topical application of these honey varieties on neutrophil recruitment to the site of arachidonic acid (inflammatory stimulus) application in a murine ear model. The results showed that several honey varieties, including Manuka honey, significantly decreased neutrophil recruitment to the site [13]. These results indicate an overall anti-inflammatory effect of Manuka honey on neutrophils, reducing their inflammatory superoxide production and attenuating their recruitment to a site of inflammation and, thus, correlate more closely than the results of the previous study with the clinical data showing that Manuka honey resolves inflammation [65,66].

Other in vivo evidence points to an anti-inflammatory effect of honey. In one example, rabbit wounds were treated with topical honey (type of honey not specified) and studied for 21 days. Histological examination of the wounds at 14 days revealed well-vascularized tissue with organized fibroblasts and collagen fibers with few inflammatory cells still present in the honey-treated group, while the non-honey group showed necrosis, uneven epithelialization, and a large neutrophil presence [66]. In a different study, Medhi et al. used a rat ulcerative colitis model to study the efficacy of rectally-applied Manuka honey to treat ulcerative colitis. Rats were administered intra-colonic 2,4,6-trinitrobenzene sulfonic acid (TBS) to induce colitis and then treated with Manuka honey at 5 g/kg body weight through a rubber tube inserted rectally. After 14 days, rats were sacrificed, and excised tissue was morphologically assessed. Histological sections of colon tissue were graded on a scale from 0 (no inflammation) to 3 (intensive inflammation). Treatment with Manuka honey decreased the sample scoring from approximately 1.8 (mean TBS control score) to approximately 0.2 (mean Manuka honey score), indicating almost no inflammation in the honey-treated samples [67]. This study indicates another promising use for Manuka honey in treating ulcerative colitis and other internal inflammatory diseases, and provides evidence of the general anti-inflammatory properties of honey which make it such an effective wound treatment. Clinical evidence also indicates that honey exhibits anti-inflammatory properties. For instance, in the 2010 diabetic foot ulcer study by Moghazy et al. that was discussed above, significantly decreased inflammation was observed in 27 of the 30 patients during and after the three-month honey treatment [51].

Together, this evidence seems to contradict the Tonks et al. study discussed above that showed that honey increases release of the inflammatory mediators TNF-α, IL-1, and IL-6 by monocytes. However, it should be noted that there are many other inflammatory and anti-inflammatory cytokines involved in the healing response that were not tested in the Tonks et al. study, and it only tested one concentration of honey rather than the gradient of honey concentrations present in a wound. Nevertheless, it is possible that these honeys cause a temporary increase in inflammatory cytokines in a wound site before later resolving that inflammation, or that by increasing inflammation the honey “shocks” the wound environment into quickly clearing infections to allow for inflammation resolution. Ultimately, future studies examining more honey concentrations and more of the relevant cytokines will be necessary to bridge the gap between these in vitro and in vivo findings. In addition, detailed time courses must be examined in order to understand the difference between how honey affects the different stages of inflammation in the wound site, especially the vast differences between the effects on the acute and chronic phases.

### 2.3. Wound Closure Effects of Honey

Studies have demonstrated that multiple varieties of honey promote wound closure. Ranzato et al. showed low concentrations (0.1% *v*/*v*) of a variety of honey types, including Manuka honey, increase closure rate in a keratinocyte scratch assay and promote fibroblast migration in a transwell insert chemotaxis assay. Specifically, 0.1% Manuka honey increased keratinocyte closure rate by 180%, and increased fibroblast migration by 150–240% (higher honey concentrations were not tested) [68]. It should be noted that this study did not make use of a sugar solution control, so it is unknown how much of this migration effect was caused by the sugar content of the honey as opposed to its other components. In the 21-day rabbit wound model study described above, faster and improved wound closure was observed in the honey-treated wounds. After 14 days, the non-honey wounds were covered by scabs and imperfect epithelialization, while skin repair in the honey-treated rabbits was perfect and detection of the injured area was difficult. Samples of the healed skin were excised and mechanically tested after 21 days, and the honey-treated rabbit skin had a significantly higher tensile yield strength (3.3 MPa) and ultimate strength (3.4 MPa) than that of the non-honey wounds (1.2 MPa and 2.3 MPa, respectively) [66]. Likewise, honey treatment in a rat dorsal wound model had similar effects. Topical application of honey to these wounds caused a 107% increase in salt-soluble collagen, a 117% increase in acid-soluble collagen, and a 109% increase in insoluble collagen after seven days relative to non-treated controls. Introduction of radiolabeled hydroxyproline one day before sacrifice allowed measurement of the collagen synthesis rate over a 24-h time period, and indicated a 124% increase in the acid-soluble collagen production rate and a 105% increase in the insoluble collagen production rate during this sixth day after wound creation relative to control, suggesting that the healing rate is increased at this time point by the honey treatment. The acid-soluble collagen extracted from honey-treated rats had a 122% increase in aldehyde content relative to that extracted from non-treated rats, indicating a higher degree of crosslinking in the wounds that were honey-treated. This was confirmed by an 11% drop in the solubility of the insoluble collagen of the honey-treated rats in the presence of urea. Interestingly, experimental groups of rats with honey administered orally and intraperitoneally showed higher degrees of collagen synthesis and crosslinking than the topical-administration group [69]. Although the administration of honey via the oral and intraperitoneal routes for wound healing has not been widely studied, these findings suggest it may, in fact, be more beneficial than the current topical administration model. The authors of this study suggest that the oral administration of the honey allows for greater nutrient uptake, which is an unsatisfactory explanation for these results as those nutrients would be processed and dispersed systemically and, thus, be unlikely to have a greater effect on the wound than topical administration. More exploration of the benefits of these routes of administration, including repeating this study, may be beneficial to confirm or disprove these potentially impactful findings.

Clinical evidence has also shown honey to improve wound closure. Numerous case studies have demonstrated beneficial effects of Manuka honey in the closure of various types of infected non-healing ulcers [70,71,72,73,74]. In the 2010 study by Moghazy et al. described earlier, ulcer size decreased in 28 of the 30 patients treated with honey, with complete healing in 13 of the patients after three months [51]. Likewise, in the study by Efem et al., it was described that the honey treatment caused more rapid wound debridement, promoted rapid epithelialization, and reduced edema, causing a faster healing rate and reduced morbidity. The author reports that within one week, sloughs, necrotic, and gangrenous tissues were separated from the ulcers enough to be lifted away by forceps without pain to the patients, while weeping ulcers were dehydrated and foul-smelling wounds were rendered odorless [53]. Unfortunately, while the progress of the wounds is described, no objective measurements of wound size or condition are included in this study, only general clinical observations of the wounds over time. In contrast, Jull et al. conducted an expansive review published in 2015 of 26 randomized or quasi-randomized trials evaluating honey as a treatment for a variety of wound types. As many of the trials examined in this review suffered from small sample sizes or reported insufficient data, few conclusions could be drawn. However, the authors did conclude that honey improves healing rates in partial thickness burn wounds relative to current gold-standard treatments, shortening healing time by about 4–5 days. They also found moderate evidence showing honey is more effective than standard antiseptic treatments in treating infected surgical wounds. However, there was insufficient evidence to make conclusions about the effects of honey in other wound types as of the publication of this review in 2015. More studies are necessary moving forward to statistically confirm the beneficial effects of honey in other varieties of wounds, such as chronically-inflamed wounds, pressure ulcers, Fournier’s gangrene, and venous leg ulcers [75].

## 3. Cautionary Evidence of Cytotoxicity

**Figure 2:**
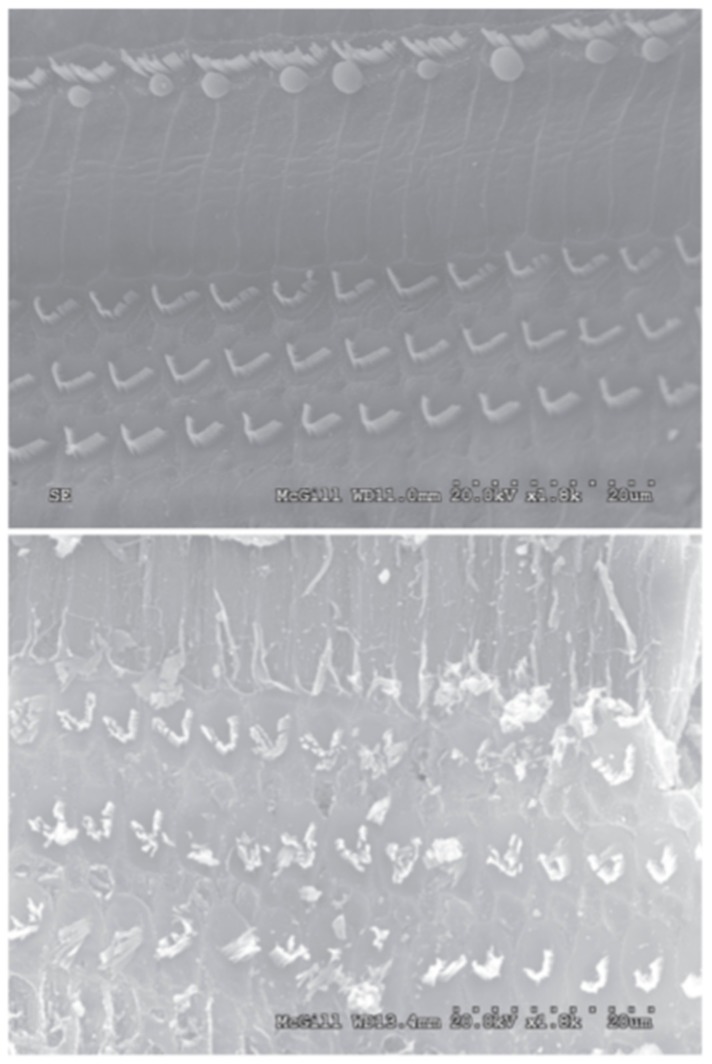
Undiluted honey damages cilia. (**Top**) SEM of saline-exposed chinchilla cochlea, in which normal inner and outer hair cells are observed. (**Bottom**) SEM of honey-exposed chinchilla cochlea in which the inner and outer hair cells have been damaged. Reproduced with permission from Aron et al., *Otolaryngology—Head & Neck Surgery*; published by BMC, 2012.

While there are many studies that have demonstrated the potential benefits of honey in wound healing, less attention has been given to the counterproductive cytotoxic effects of high concentrations of honey. However, a few groups have studied these cytotoxic effects in various cell and animal models, and their data provides a cautionary window into the dangers of using high concentrations of honey in wounds or other therapeutic applications. An in vitro study by Sell et al. found that honey concentrations of 5% *v*/*v* or above were cytotoxic, killing almost 100% of the cells tested in fibroblast, pulmonary microvascular endothelial, and macrophage cultures after one day [65]. For reference, the Sherlock et al. study referenced earlier showed little inhibition of MRSA, *E. coli*, or *P. aeruginosa* growth at concentrations in the range of 6–12% *v*/*v* or below [41]. Marckoll et al. also found a minimum inhibitory concentration of Manuka and Norwegian Forest honey on a variety of bacterial biofilms to range between 6–12% for Manuka honey and 12–25% for Norwegian Forest honey. The cytotoxicity of honey has also been studied in vivo. A study in which 50% *v*/*v* Manuka honey was applied to chinchilla ears found that it caused severe inflammation and ototoxicity. Eight chinchillas had the honey solution applied to the round window membrane and the cochlea of one ear while the other ear received a sham treatment of normal saline solution. All eight chinchillas developed a head tilt and facial paralysis on the side of the experimental ear within 0–48 h of honey application, with a corresponding loss of balance and nystagmus. Extraction of the osseous bullae and cochleae showed that the honey-exposed bullae were soft and brittle and the cochleae were darker, compared to the control bullae and cochleae which were normal in color and consistency. Histological examination revealed a scarcity of cells and the creation of new vacuoles within the honey-exposed spiral ligaments, with damage to the organ of Corti and an excess of inflammatory cells found in the honey-exposed cochleae, whereas the saline-treated organs had normal appearance, architecture, and cellularity. Scanning electron microscope (SEM) images showed severe damage to the spiral ganglion and cochlear hair cells in the experimental ears, with no damage to the control ears, as shown in Figure 2 [76]. Possible causes of this damage are the low pH of the honey and its high osmolarity, although further testing with other acidic and hypertonic solutions will be necessary to confirm this theory. In a similar in vivo effort, Paramasivan et al. flushed ovine frontal sinuses with methylglyoxal concentrations ranging from 0.5 to 7.2 mg/mL, or with 16.5% *w*/*v* Manuka honey enriched with methylglyoxal in the same concentration range, twice daily for 14 days. Animals were sacrificed, and the tissue was analyzed by histology and tested for *S. aureus* biofilms which had been intentionally developed in the ovine sinuses before the study. The results indicated both the methylglyoxal alone and 16.5% Manuka honey enriched with methylglyoxal above 0.9 mg/mL eradicated the *S. aureus* biofilms, while honey/methylglyoxal treatment with less than or equal to 1.8 mg/mL methylglyoxal was non-irritating to the mucosa. However, methylglyoxal and honey/methylglyoxal treatment with methylglyoxal levels above 1.8 mg/mL caused cilia denudation and squamous metaplasia, indicating tissue damage [77]. These results point to methylglyoxal as a culprit of Manuka honey’s cytotoxicity, although more testing in a variety of cell and animal models should be done to confirm this finding. If accurate, however, these results could indicate that other honey varieties without methylglyoxal may be optimal for applications in which a high honey concentration would be required.

The results of these papers provide compelling evidence of the cytotoxicity of Manuka honey at higher concentrations, and should prompt a re-assessment of the current use of Manuka honey in wound treatment. Since most clinical honey treatment involves directly applying undiluted honey to the wound, it is likely that honey’s beneficial effects are at least somewhat counteracted by its cytotoxicity. Even accounting for dilution of the honey by wound exudate and excess liquid pulled from the deeper tissue by honey’s high solute osmolarity, cells within the wound likely encounter honey concentrations at or above the 5% *v*/*v* cytotoxic concentration found by Sell et al. [65]. When designing tissue regeneration templates, this cytotoxicity must be accounted for to avoid killing infiltrating cells and impeding tissue ingrowth. Even in applications where tissue ingrowth is not necessary, such as a bone screw, care must be taken to avoid causing necrosis in the surrounding area. Thus, it is paramount that these tissue templates and devices that incorporate honey do so in a way that allows a low-level, controlled release to avoid cytotoxicity while prolonging the beneficial effects of the honey. However, as described above, higher levels of honey are necessary to impede or destroy bacterial biofilms. Therefore, in situations where a bacterial biofilm has been established, it may be necessary to first treat the wound with undiluted honey to eradicate this biofilm. Once the biofilm has been eliminated, a template can be applied that releases lower levels of honey over time to reduce inflammation and induce tissue regeneration and infiltration. It should be noted that certain beneficial effects discussed earlier, such as immunomodulation and promotion of wound closure, occur at honey concentrations below the cytotoxic level. However, the data does not currently exist to definitively state a therapeutic window for each of the beneficial effects of the honey discussed earlier. As such, the authors recommend that testing for cytotoxicity and desired therapeutic effects (fibroblast infiltration, immunomodulation, etc.) be performed for each honey-incorporating template using assays that take into account the expected microenvironment of the template upon implantation.

## 4. Honey in Tissue Engineering

Given the amount data supporting the use of honey as a wound treatment, the logical next step is to apply these findings to the field of tissue engineering and biomaterials. The implantation of a biomaterial within the body necessitates the creation of a wound, and the presence of these biomaterials provides a potential site for bacteria to deposit and fester after implantation. The antibacterial effects of honey, especially Manuka honey, could significantly reduce the rates of infection in biomaterials. Additionally, given the evidence showing that Manuka honey reduces inflammation and promotes fibroblast migration and collagen deposition, it is likely that it could promote tissue-material integration/regeneration and accelerate healing of the surrounding wound site [13,65,66,68,69]. An important consideration will be how to apply the honey to the biomaterial or incorporate it into the biomaterial to deliver appropriate concentrations of honey and achieve these optimal effects. It is likely that in most applications in which a bacterial biofilm is not present, a controlled-release profile will be desirable to avoid cytotoxic effects and prolong the presence of the honey in the region of interest/need. Thus, research has been focused into incorporating the honey throughout biomaterials to achieve this type of release. In the past decade, there have been numerous studies incorporating honey into a variety of biomaterial tissue templates for tissue regeneration.

### 4.1. Electrospun Templates

One of the first attempts to incorporate Manuka honey into an electrospun template was published in 2012 by Vadodaria et al. In this study, Manuka honey was combined with polyethylene oxide (PEO) into solutions which were then electrospun. SEM images showed that increasing Manuka honey content caused thicker, merged fibers, although these morphological changes could be somewhat compensated for by reducing the solution feed rate and increasing the applied voltage. Fourier-transform infrared spectroscopy (FTIR) revealed peaks indicating the presence of both PEO and Manuka honey in the fibers, while differential scanning calorimetry (DSC) indicated that increasing the Manuka honey content lowered the melting point of these templates [78]. This study did not include any experiments examining biocompatibility or cell behavior on the templates and did not include a honey release profile. Nevertheless, it established the basic parameters necessary for electrospinning Manuka honey into nanofibrous templates for use as delivery vehicles.

In another early study, Maleki et al. electrospun poly(vinyl alcohol) (PVA) templates containing various concentrations of Iran-Tabriz honey and dexamethasone, an anti-inflammatory drug. Both honey and dexamethasone decreased the fiber diameters of the templates in a dose-dependent fashion. Dexamethasone release studies showed a large burst release of dexamethasone within the first 10 min of soak due to the swelling of the PVA fibers, with no difference between the honey and non-honey dexamethasone templates [79]. Like the Vadodaria et al. paper, this effort did not conduct any cellular studies or bacterial inhibition assays, but it does indicate that honey can be incorporated into electrospun templates along with other additives. Unfortunately, although this paper did have release profiles of the dexamethasone from the templates, no honey release profile was included.

**Figure 3:**
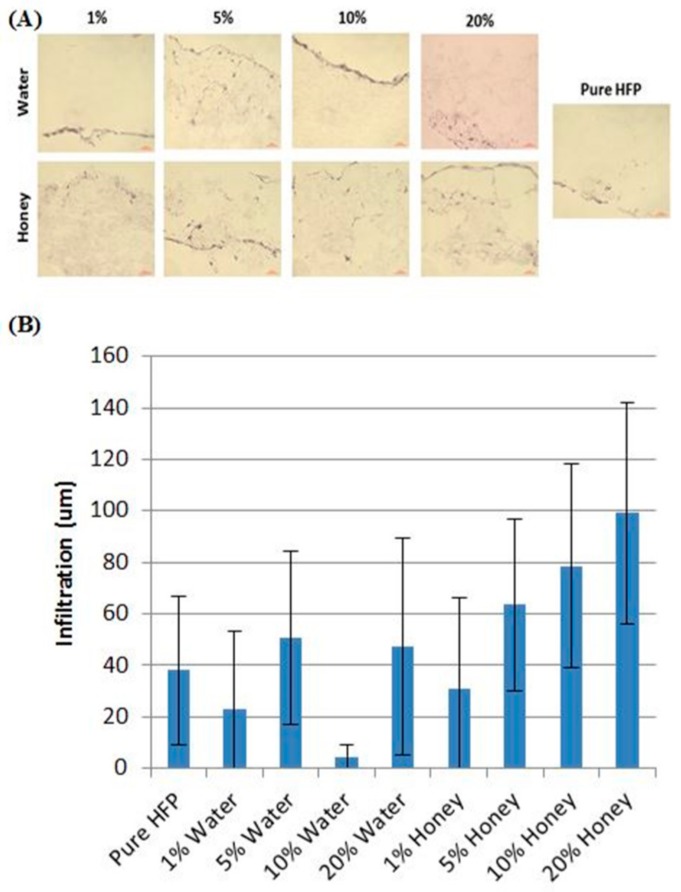
Honey induces fibroblast infiltration. (**A**) Representative images of H and E-stained honey templates and water controls after 28 days of fibroblast culture. (**B**) Cellular infiltration depth of the furthest 60 cells on each image. Reproduced with permission from Minden-Birkenmaier et al., *Journal of Engineered Fibers and Fabrics*; published by INDA, 2015.

A more in-depth study of Manuka honey in electrospun templates was published by Minden-Birkenmaier et al. in 2015. In this study, solutions of poly(ε-caprolactone) (PCL), 1,1,1,3,3,3-hexafluoro-2-propanol (HFP), and various concentrations of Manuka honey were electrospun into fibrous templates which were then characterized with regards to fiber morphology, water vapor transmission rate (WVTR), permeability, mechanical properties, honey release, fibroblast response, and bacterial inhibition. Templates were also created replacing the Manuka honey with equivalent amounts of water to use as morphological controls. By sonicating the Manuka honey in the HFP before adding the PCL, and then electrospinning the resulting solution within 24 h, templates were created with equivalent fiber diameters, varying from 2 µm to 3.5 µm in diameter, up to a 20% *v*/*v* honey content. The water vapor transmission rate after a one-hour soaking period increased with increasing honey content, as did template permeability. Honey incorporation caused a decrease in the elastic moduli and peak stress of the templates, but there was no significant change in these properties over a 28-day soaking period. Glucose assays indicated that while up to 80% of the honey content of the templates was lost during a one-hour ethanol disinfection soak, the templates retained enough honey that they released significant amounts of honey over the following 24-h period of soaking, proportional to the amount incorporated into the scaffold. Fibroblast chemotaxis assays showed no effect of honey content in inducing chemotaxis towards the templates, indicating that the honey left in the templates to be released after the disinfection step is probably too low of an amount to induce chemotaxis. However, the 20% honey templates caused a significant increase in fibroblast proliferation and infiltration over PCL controls, indicating the potential of Manuka honey to improve template-tissue integration and regeneration (Figure 3). Bacterial inhibition studies of *E. coli* and *S. agalactiae* showed significant inhibition of both bacterial types by the 10% and 20% honey templates as expected due to the antibacterial properties discussed earlier, although this inhibition was significantly less than that of a sterile disc swabbed with pure Manuka honey. Together, these findings indicate the potential benefit of Manuka honey in improving cellular proliferation, cellular ingrowth, and bacterial inhibition associated with a tissue template [80]. However, the fact that 80% of the incorporated Manuka honey was removed during ethanol disinfection indicates that future studies may consider investigating core-shell electrospinning to protect the honey from leaching out during disinfection and provide for a more long-term, controlled release period. Other disinfection or sterilization methods, like gamma irradiation, should also be investigated as alternatives that may remove less of the incorporated honey.

The physical properties detailed in the Minden-Birkenmaier et al. study also indicate that the majority of the honey is likely sequestered to the fiber surface during the electrospinning process, where it is easily released once rehydrated, allowing for greater template permeability, but maintaining mechanical strength due to the PCL fiber core [80]. As expected, this morphology makes the templates hygroscopic, allowing them to soak up water, but changing their physical properties as they are exposed to ambient humidity or liquid water (as demonstrated by the water vapor transmission rate data discussed above). It will be important to take this hygroscopidity and the associated processing issues into account when producing and packaging honey-laden templates for clinical use. For instance, production and packaging in a low-humidity environment may be necessary to improve the shelf-life of future commercial honey-laden templates.

In a more recent effort, Balaji et al. combined Malaysian Tualang honey and papaya extract (PA) (also reported to have antimicrobial and anti-inflammatory properties [81,82]) into a *N*,*N*-dimethylformamide (DMF) solution along with polyurethane (PU), which was then electrospun into templates. Fiber diameter measurements showed that the honey and papaya extract reduced the template fiber diameter, from a mean diameter of 434 nm for PU controls down to 190 nm for the PA/honey templates; however, porosity only experienced a minor decrease. FTIR confirmed the presence of both the honey and the PA along with the PU in the fibers. Water absorption tests revealed that the presence of both honey and PA in the templates caused a three-fold increase in water uptake, indicating a potential benefit of these hydrophilic substances in absorbing wound exudate. Hemocompatibility studies demonstrated that the PA/honey templates had significantly greater adsorption of albumin, but significantly less adsorption of fibrinogen relative to PU controls, indicating a resistance to clotting. Activated partial thromboplastin (APTT) and prothrombin time (PT) assays likewise demonstrated that the PA/honey templates took longer to activate thromboplastin and prothrombin than the PU controls, 180 s (honey) versus 152 s (control) for APTT and 45 s (honey) versus 37 s (control) for PT. The PA/honey templates also had a decrease in the hemolytic percentage from the PU controls (2.7% for PU control and 0.9% for the honey template) indicating a reduction in red blood cell lysis. This hemocompatibility suggests a possible use of the template in vascular tissue engineering [83]. While the results of this study are impressive, particularly with regards to hemocompatibility, it would have been beneficial for separate templates containing different amounts of honey or PA to be tested, as the effects of the honey and the PA on hemocompatibility could have been isolated from each other. This separation of honey and PA would have allowed for a more robust study to indicate the true hemocompatibility potential of the honey and the PA alone.

Several studies have investigated combinations of silk fibroin and honey in electrospun templates. Kadakia et al. electrospun silk fibroin templates from HFP containing either poloxamer 407 (P407), a hydrophilic polymer used to improve cell adhesion, or Manuka honey. Fiber diameter measurements taken from SEM images revealed that the incorporation of P407 at either a 1:1 or 3:1 silk:P407 ratio (total polymer concentration of 10% *w*/*v*) significantly decreased fiber diameters (from 2.2 μm down to 1.8 μm), while 1% honey increased fiber diameters (from 4.4 μm up to 5.8 μm) and 5% honey decreased fiber diameters (down to 3.6 μm). Mechanical testing showed that increasing P407 and honey concentrations decreased the elastic moduli of the templates relative to silk control templates when dry. However, when the templates were hydrated, the honey templates had elastic moduli in the range of 5–9 MPa, above the 2–3 MPA range of the silk controls, indicating that the honey increased elasticity. While no difference was observed in the swelling of the silk/P407 templates and pure silk controls, the honey templates swelled to a significantly higher degree, with a swelling ratio of about 350% after four hours, while the silk fibroin control had a swelling ratio of approximately 240%. The water vapor transmission rate was observed to decrease from approximately 1750 g/m^2^/day for the silk control template to approximately 1550 g/m^2^/day with the incorporation of 1% honey and approximately 1400 g/m^2^/day with the incorporation of 5% honey. Water contact angle measurements showed that the incorporation of p407 decreased the water contact angle from about 70° to about 45° for 3:1 silk:P407 and about 11° for 1:1 silk:P407. Surprisingly, the incorporation of honey increased the water contact angle from around 61° to approximately 67° for 1% honey and approximately 78° for 5% honey. Fibroblast experiments showed no increase in proliferation in the 1% honey templates, and impeded proliferation on the 5% honey templates, indicating a degree of cytotoxicity of the honey as discussed earlier in this review. However, fibroblasts infiltrated fully into all template types after 28 days, with no differences between groups, and no significant difference in hydroxyproline production was observed between the groups [84]. This study would have benefited from a glucose release assay to ascertain the amount of honey released during disinfection and subsequent culture. Given the cytotoxicity observed in the fibroblast culture experiments, it is speculated that templates with lower amounts of loaded honey or a lower, persistent release of honey over time would have performed better in the cellular studies.

**Figure 4:**
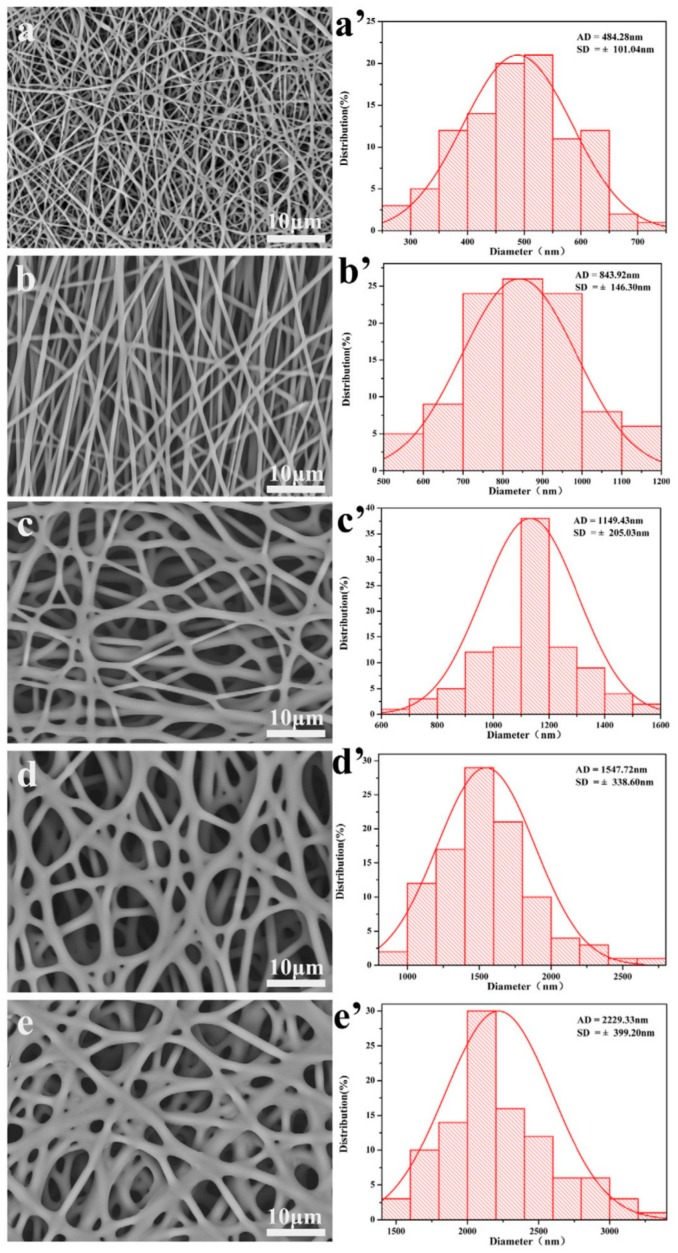
Honey increases fiber diameter. SEM images and histograms of fiber diameters of silk/PEO nanofibrous matrices spun with (**a**,**a’**) no Manukah honey; (**b**,**b’**) 10% *w*/*v* Manuka honey; (**c**,**c’**) 30% *w*/*v* Manuka honey; (**d**,**d’**) 50% *w*/*v* Manuka honey; and (**e**,**e’**) 70% *w*/*v* Manuka honey. Reproduced with permission from Yang et al., *Materials & Design*; published by Elsevier, 2017.

In a similar effort, Yang et al. electrospun solutions of silk fibroin and poly(ethylene oxide) (PEO) with concentrations of 0%, 10%, 30%, 50%, and 70% *w*/*v* Manuka honey into nanofibrous templates. FTIR showed the presence of the Manuka honey in the fibers, and SEM images showed an increasing fiber diameter with honey concentration, from an average of 484 nm without honey to an average of 2229 nm with 70% *w*/*v* honey, as shown in Figure 4. Bacterial inhibition tests using *E. coli*, *S. aureus*, *P. aeruginosa*, and MRSA indicated that the templates retained the antimicrobial effects of the Manuka honey. Specifically, bacterial inhibition over 24 h of all four bacterial strains was approximately zero for the non-honey template, but increased to around 50% inhibition of *E. coli*, about 28% inhibition of *S. aureus*, about 57% inhibition of *P. aeruginosa*, and about 40% inhibition of MRSA for the 70% *w*/*v* honey template. Templates were also used to treat a mouse dorsal wound model over a 12-day period, and showed complete healing of the wounds treated with the 70% honey template, whereas wounds treated with a non-honey silk template or a commercial AquacelAg wound dressing had only around a 90% reduction in wound size over this timeframe [85]. The most novel part of this study was the use of deionized water and hydrophilic polymers in the electrospinning process, as opposed to the organic solvents used in the previously described studies. This water-based solution could potentially eliminate the sequestering of the honey to the outside of the fibers, reducing the mechanical strength of the template, but delaying the release of the honey over time. As such, this study would have benefited from a glucose release experiment showing the release profile of honey from the template to indicate if this delayed, controlled release is present.

### 4.2. Cryogels

Cryogels, fabricated by freezing a crosslinked polymer solution, have been investigated as templates for bone tissue engineering due to their porosity, elasticity, and ability to retain their three-dimensional architecture. As bone fractures or defects are often sites of biofilm formation and bacterial infection due to their open nature, the eradication of bacteria is of utmost importance. Thus, research has focused on incorporating honey into cryogels as an antimicrobial agent. In a 2017 study, Hixon et al. incorporated Manuka honey into cryogels formed from either gelatin or silk fibroin. While the silk cryogels had larger pores (average pore diameter 25–40 μm) than the gelatin cryogels (average pore diameter 17–20 μm), the incorporation of Manuka honey significantly decreased these pore diameters in the silk cryogels, but not in the gelatin cryogels. Honey decreased the swelling ratios of the gelatin cryogels, but not the silk ones. Ultimate compression testing indicated that honey significantly decreased the average peak stress in both cryogel types, and decreased the modulus of the gelatin cryogels, which could make the honey-incorporated cryogels less feasible in load-bearing bone tissue applications. Manuka honey incorporation had no significant effect on the proliferation of seeded MG-63 osteosarcoma cells, but increased cellular infiltration in the highest (10% *v*/*v*) honey concentration silk cryogel samples. Similar to the results observed in the electrospun honey templates discussed above, glucose release tests showed the bulk of the incorporated honey was released within the first hour of hydration, however, after this bulk release there was a consistent release of 0.03 mg/mL glucose per day throughout the 14-day soak period for both the gelatin and silk cryogels. The peracetic acid sterilization procedure also was shown to remove most of the incorporated honey from both polymer types. Thus, it may be beneficial to use other sterilization methods, such as gamma radiation, in the future to avoid leaching out the honey from these structures. Bacterial clearance tests showed that the incorporation of honey significantly increased bacterial clearance of both *E. coli* and *S. agalactiae*, and bacterial broth clearance and bacterial adhesion assays confirmed this trend. Honey incorporation did not alter mineralization of the cryogels by the MG-63 cells over a 28-day culture period [86]. While the mechanical testing data indicates that the presence of honey weakens these cryogels and makes them more brittle, their ability to inhibit bacterial growth and induce cellular infiltration suggests their potential usefulness in bone tissue engineering. This tradeoff of mechanical stability should be accounted for when designing future honey cryogel-based therapies. Efforts should focus on protecting the honey content from washing out during sterilization or as an initial bulk release, creating a more long-term, sustained release greater than one-to-two hours. Additionally, the utilization of other polymers should be explored as a means to maintain mechanical strength and elasticity even with the incorporation of honey.

Although not addressed in this study, it has been shown that lowering the pH in the area around bone tissue can stimulate increased bone resorption and reduce mineral deposition by osteoclasts [87,88]. This effect is absent at or above a pH of 7.4, but is near-maximal at a pH of 7 [88]. Thus, there is a danger that the low pH of honey could impede bone regeneration rather than stimulate it. The study by Hixon et al. showed no effect of the honey on MG-63 osteosarcoma cell mineralization in vitro, but additional testing with non-cancerous osteoblasts and osteoclasts should be done. Given these well-documented effects of lowering the pH on bone resorption, it is speculated that honey-incorporated cryogels are may not be a useful bone-repair therapy.

In a subsequent study by Hixon et al., Manuka honey with various UMFs (unique manuka factor, a general quantification of bacterial inhibition) was incorporated into cryogels and electrospun templates, both fabricated from silk fibroin. The amount of honey in all constructs was kept constant at 5% *v*/*v*, while the UMF was varied by utilizing commercially available honeys rated with UMFs of 5+, 10+, 12+, 15+, and 20+. In general, UMF had no effect on the morphology of the cryogels or electrospun templates or on their ability to inhibit *E. coli* or *S. aureus*, and the electrospun templates had greater bacterial clearance (0.5–1 cm) than the cryogels (approximately 0.16 cm). The glucose release profiles from the cryogels and the electrospun templates were not statistically different, with the bulk of the glucose released within the first four days of soak maintaining a level of 0.4–0.6 mg/mL glucose in the surrounding solution [89]. Thus, it is unknown why the electrospun templates were more effective at clearing both types of bacteria. One explanation could be that different bactericidal components of the honey, such as the methylglyoxal, hydrogen peroxide, or the gluconic acid, are released at different rates or profiles than the glucose, and these rates may be different between the cryogels and the electrospun templates. Assays for these other components may be necessary to fully explain the dramatic difference in bacterial inhibition between these template types. Likewise, it is curious that the UMF of the honey used in these templates did not affect their bacterial clearance. Part of the problem may be that the exact UMF of each honey obtained was not listed, only that it was above the listed level (5+ means that it has a UMF of at least 5, not exactly 5). Thus, it is possible that the actual UMFs of the honeys did not vary as much as was thought based on their labels. It would be beneficial for future studies testing different UMFs to test the UMF in-house, rather than relying on the UMF rating of the commercial vendor. Thus, it is still unknown whether the UMF of the Manuka honey used affects bacterial clearance or other properties when incorporated into tissue templates.

### 4.3. Hydrogels

**Figure 5:**
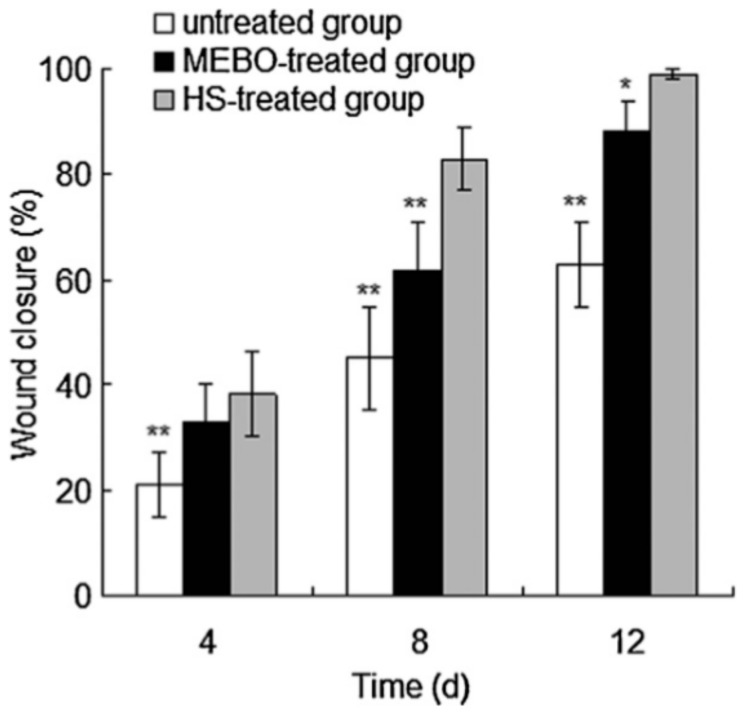
Honey hydrogel induces wound closure. Wound closure rates in rabbit wounds that were untreated, treated with a commercial wound ointment (MEBO), or treated with a honey-infused chitosan/gelatin hydrogel (HS) at 4, 8, or 12 days after beginning of treatment. “**” indicates statistical significance at *p* < 0.005, “*” indicates statistical significance at *p* < 0.01. Reproduced with permission from Wang et al., *Carbohydrate Polymers*, published by Elsevier, 2012.

Hydrogels, highly absorbent networks of hydrophilic polymer chains, are often used as templates and drug delivery devices in tissue engineering due to their polymeric structure and the ability to control characteristics, such as pore size, water content, and degradation profile. Several groups have explored incorporating honey into hydrogels for use as wound coverings. In a 2012 study, Wang et al. incorporated Chinese Sunflower honey at 10% or 20% *v*/*v* into hydrogel sheets fabricated from chitosan and bovine gelatin. Swelling studies indicated that the presence of honey reduced the ability of the hydrogel to absorb fluid, with the 20% honey hydrogels swelling only around 250%, as compared to the 700% swelling of the non-honey control. Compression testing indicated that honey content also reduced the modulus of the hydrogel sheets from around 110 kPa to around 60–70 kPa for the 20% honey hydrogel and approximately 58 kPa for the 10% honey hydrogel. Antibacterial assays showed that the presence of honey significantly increased the inhibition of *S. aureus* and *E. coli* growth, with the 20% honey hydrogel causing almost 100% inhibition while the non-honey hydrogel caused approximately 20% inhibition of both bacterial types. In vivo oral toxicity tests were performed in mice, and dermal irritation and burn wound healing tests were performed in rabbits. As expected, the mice toxicology tests showed no toxic symptoms. After eight days of treatment in rabbit wounds, the honey hydrogel group averaged about 80% wound closure, while the ointment group had about 60% wound closure and the non-treated group had about 45% wound closure, as shown in Figure 5. Histological examination of the wounds after 12 days revealed that the untreated wounds were infected, contained a high amount of inflammatory cells, and had no hair follicles. The ointment-treated group had smaller ulcers than the non-treated group, but still contained acute inflammatory infiltrate that collected in small cysts underneath the regenerated epidermis. Both the ointment group and the honey hydrogel group showed epidermal healing, but the honey hydrogel group had less inflammatory infiltrate and also had proliferating hair follicles on the surface [90]. Although this study thoroughly characterized the in vitro and in vivo aspects of the honey hydrogel, the lack of a control non-honey hydrogel group in the animal studies call into question whether the honey improved the in vivo performance of the hydrogel. Additionally, the lack of a topical honey treatment group in these studies made it impossible to ascertain whether incorporation of the honey into a hydrogel improved healing over the current clinical method of treatment. Thus, the only conclusions that can be drawn from this section of the study are that this honey-containing hydrogel improves healing relative to an ointment treatment. A glucose release study would also improve this paper by showing whether the honey is released in a burst or released in a controlled fashion over days or weeks after implantation.

More recently, Sasikala et al. incorporated Manuka honey into chitosan hydrogel films, also for use as wound dressings. Chitosan solutions containing 8% *w*/*v* Manuka honey were cast in Petri dishes and dried at 40 °C for 24 h. Honey increased the folding endurance of the samples with the honey samples surviving a mean of 289 folds, whereas the non-honey films survived a mean of 143 folds. This finding indicates greater flexibility of the honey hydrogels, which is likely a function of the hygroscopic effect of the honey. However, no effect of the honey was observed on the water vapor transmission rate of these films, which was curious given the results observed in electrospun scaffolds as detailed earlier in this review. As observed in the study discussed above, honey decreased the swelling ratios of the hydrogel films and increased the inhibition of *S. aureus* and *E. coli* growth. When these films were placed in a rat dorsal wound model, increased wound closure was observed in the honey samples relative to non-honey control films and cipladin ointment controls. Specifically, after 12 days of treatment, the honey hydrogel wound was 94% closed, the non-honey hydrogel wound was 78% closed, the ointment-treated wound was 86% closed, and the non-treated control wound was 64% closed [91]. The use of non-honey control hydrogel films in this animal study show the benefit of the honey to the wound healing process, which is an improvement over the Wang et al. study discussed above. However, many of the studies described in this paper seem to have been undertaken with a sample size of *n* = 1, as there are no standard deviations or standard errors reported. Although the methods section says that an ANOVA was performed on the wound closure data, it does not report which specific sample groups were significant from each other, casting doubt as to the scientific veracity of these findings. This study would benefit from being repeated more thoroughly so that its findings can be scientifically corroborated.

In 2008, Gethin et al. published a study in which 20 patients with chronic, non-healing leg ulcers were treated with Apinate, a commercially available Manuka honey hydrogel dressing made by Derma Sciences. This study focused on the effect of the Manuka honey hydrogel in lowering the wound pH, and the corresponding effect on wound size reduction. The Apinate dressing itself had a pH of 4.0, due to the acidity of the honey. After a two-week period, wounds treated with Apinate had a mean pH drop of 0.46, with a mean wound size reduction of 1 cm^2^. A linear regression model was developed using the experimental data, showing a significant relationship between drop in pH and a reduction in wound size over the two-week period, with a one unit reduction in pH being associated with a decrease of 81% of wound size [3]. It is unknown how much of the healing effect was a function of the honey’s pH, as opposed to its osmotic effects, bactericidal effects, or other properties detailed earlier in this review, or whether the pH is an effect of wound healing instead of a cause. However, a decrease in pH has been shown to increase oxygen saturation, reduce elastase activity, and kill certain bacteria, which all aid wound healing [92,93,94].

**Figure 6:**
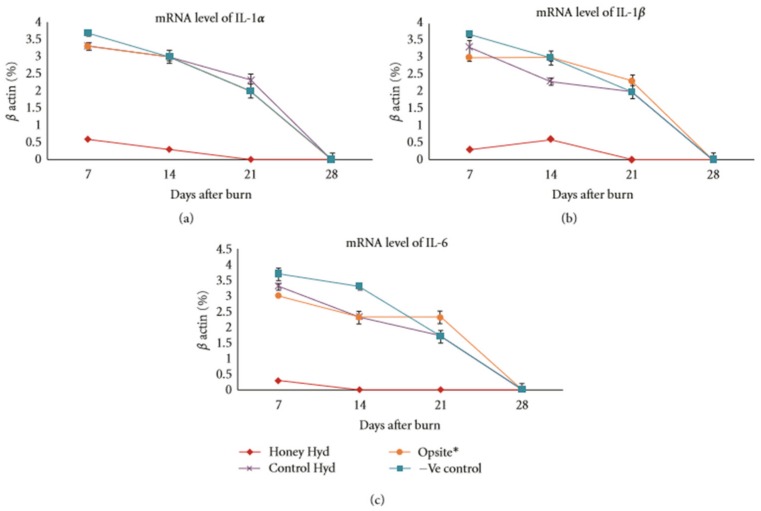
Honey reduces inflammatory cytokine expression. mRNA expression of (**a**) IL-1α, (**b**) IL-1β, and (**c**) IL-6 in a rat burn wound model treated with a control hydrogel, a commercial Opsite film dressing, the honey hydrogel, or non-treated (-Ve) control, normalized to β-actin. Reproduced with permission from Zohdi et al., *Evidence-based Complementary and Alternative Medicine*; published by Hindawi, 2012.

Giusto et al. have conducted research incorporating Manuka honey into pectin-based hydrogels. They report that honey-containing pectin hydrogels have superior bacterial clearance of *S. aureus* and *E. coli*, and demonstrate no cytotoxicity to fibroblasts [95,96]. Another study was conducted by Zhodi et al. in which Gelam honey, a honey produced in Malaysia, was incorporated into hydrogels made from polyvinyl pyrrolidone (PVP) and polyethylene glycol (PEG). Honey content significantly decreased the pH value of the hydrogels (from 5.3 to 4.3) and increased the swelling of the hydrogels by a factor of five relative to non-honey controls. A large-scale burn wound study was undertaken using 96 rats, six rats per experimental group. Wounds treated with the honey-containing hydrogels significantly decreased in size relative to non-honey hydrogel controls by days 21 and 28, with the honey-treated wounds averaging a 91% reduction in size compared to the 72% reduction in size of the control hydrogel wounds. Histological examination showed decreased inflammatory exudate by day seven and increased dermal repair and reepithelialization by day 21 in the honey-containing hydrogel wounds. These wounds also showed an increase in granulation tissue and capillary formation, as well as collagen synthesis. RNA extracted from the wound site showed that the honey-containing hydrogel treatment caused a significant decrease in IL-1α, IL-1β, and IL-6 expression relative to control hydrogels, a commercial Opsite film wound dressing, or non-treatment groups. Specifically, the honey hydrogel caused a drop from around 3.5% expression to 0.5% of IL-1α and IL-1β, and from about 3.5% expression to 0.1% expression of IL-6 mRNA after seven days, normalizing expression to a β actin control, as shown in Figure 6 [97]. This animal study is the most in-depth look at the in vivo effect of honey-containing hydrogels and demonstrates that the honey content reduces inflammatory cytokine output, reduces inflammatory exudate, increases the formation of granulation tissue, and increases the wound closure rate. Ideally, future studies of this type will also look at a greater number of relevant secreted factors, such as TNF-α, IL-8, MIP-1α, MIP-3α, VEGF, MMP-1, MMP-9, and Proteinase 3, among others. In this way, a more complete understanding of the wound environment could be ascertained.

## 5. Commercialization

Currently, several companies sell or are developing products which contain Manuka honey for wound care, as shown in Table 2. Derma Sciences, a tissue regeneration company based in Princeton, New Jersey, sells a line of Manuka honey products under the brand Medihoney^®^. In addition to pastes and gels combining Manuka honey with gelling agents to increase viscosity, this company also sells several variants of an alginate-based hydrogel sheet containing Manuka honey for use as wound coverings, including the Apinate dressing discussed earlier in the hydrogel section [98]. Several in vitro studies have confirmed the antibacterial effects of these products [99,100]. Furthermore, randomized controlled trials and case studies have demonstrated the antibacterial and wound healing effects of these products in the clinical setting [73,101,102,103,104,105]. Simon et al. detailed the treatment of various surgical wounds and drainage sites in pediatric oncology patients with a Medihoney^®^ alginate wound covering, and reported that the honey reduced irritation and cleared infections. One acute lymphatic leukemia patient with a high level of immune suppression had a persistent deep surgical site infection that healed completely once treated with the Medihoney^®^ [73]. Johnson et al. conducted a randomized controlled trial of topical Medihoney^®^ application versus mupirocin in preventing catheter-associated infections and found that while the honey was comparable to the mupirocin in preventing infection, 2% of staphylococcal isolates were mupirocin-resistant. Thus, they concluded that honey represented a good alternative to the gold-standard antibiotic [101]. A wound-focused randomized controlled trial conducted by Robson et al. using topical Medihoney^®^ observed about a 10% increase in the healing rate in the honey-treated wounds as opposed to conventionally-treated wounds, which was statistically significant [102]. A prospective observational study by Biglari et al. using Medihoney^®^ focused specifically on chronic pressure ulcers and found that the honey eradicated bacterial growth in all 20 ulcers treated, with 90% of patients showing complete wound healing after four weeks [104]. Likewise, Smith et al. published a case series of topical Medihoney^®^-treated recalcitrant venous leg wounds that had failed to respond to four-layer compression, topical silver, nonadherent dressings, and antibiotic therapy. All 11 wounds treated had 100% closure by six weeks, with an average wound healing velocity of 0.25 cm^2^/day [105]. In addition to proving the efficacy of the Medihoney^®^ product line, specifically, these trials and case studies lend additional weight to the general benefits of Manuka honey on wounds.

In addition to the Medihoney^®^ line by Derma Sciences, there are a few other companies that sell Manuka honey-based therapeutic products. The New Zealand-based Manuka Health (Newmarket, Auckland) manufactures a glycerin-based honey-containing hydrogel sheet for wound covering [106]. Another New Zealand-based company, ManukaMed Ltd. (Solway, Masterton), sells variations of gauze-based, honey-impregnated fiber pad wound coverings [107]. The United Kingdom-based Advancis Medical (Kirkby-in-Ashfield, Nottinghamshire) makes several types of Manuka honey-impregnated wound coverings, including cellulose-based net dressings (Actilite and Activone Tulle) and alginate hydrogels (Algivon, Algivon Plus) [108]. Additionally, a Memphis, Tennessee-based company, SweetBio Inc. (Memphis, TN, USA), is developing a resorbable membrane for oral surgery, with manufactured prototypes currently undergoing testing [109]. Of these products, the only ones extensively studied in peer-reviewed literature are the Medihoney^®^ coverings. Except the Sweetbio membrane, all of these products are designed as temporary wound coverings that must be physically replaced. While no company has published release profiles of the honey from their products, given the described methods of application it is likely that the honey is delivered at high concentrations as a bolus. As high concentrations of Manuka honey have been observed to be cytotoxic in vitro, there may be a potential to improve the healing outcomes of wounds treated with these products by diluting the honey or by attenuating the release to lower levels for prolonged amounts of time [13]. However, attenuating the honey concentration may diminish the antibacterial effects of the dressings. Thus, higher concentrations of honey should be used initially in infected wounds to ensure the killing of the invading bacteria. A two-step process may be optimal, in which heavily infected wounds are first treated by topical application of undiluted Manuka honey to eradicate the infection, and then the honey is removed and replaced by a controlled-release template to aid in tissue regeneration. Alternatively, delivery of lower levels of honey alongside an antibiotic could enable the eradication of bacteria and stimulation of healing without the undesirable cytotoxic effects of the honey.

## 6. The Future of Honey in Tissue Engineering

The greatest hurdles to be overcome in the development of honey-containing tissue engineering templates are the cytotoxicity of high concentrations of honey and the lack of prolonged, consistent release rates of the honey over time. In templates which rely on cellular infiltration and tissue ingrowth, cells may encounter higher concentrations of honey as they infiltrate into the honey-containing template than they would outside or adjacent to that template. Such templates will be surrounded by a honey gradient radiating away from them into the surrounding tissue, and migrating cells will encounter higher and higher honey levels as they move towards, and into, the template. Thus, it will be important to monitor honey levels not only in the template releasate, but within the template environment itself during in vitro honey release studies. While some of the studies discussed above did not see honey cytotoxicity as an impediment to cellular proliferation and infiltration, these studies used liquid ethanol disinfection or peracetic acid sterilization steps that washed away the majority of the honey from the templates before cell seeding [80]. In applications where templates are disinfected via ultraviolet or gamma radiation, or ethylene oxide sterilization, no honey will be removed before cell seeding or template implantation. Thus, cytotoxicity could impede cell infiltration and proliferation. The templates studied thus far tend to release their honey content in a bolus during the first day of soak or implantation. While this type of release may be acceptable for a wound covering that can be removed and replaced, templates that are surgically implanted and resorbed into the body must contain the entire honey amount necessary for the application. Thus, it is important that methods be developed to attenuate and delay the release of honey over a period of days to weeks. In electrospun fibers, this may be accomplished by the use of core-shell electrospinning, in which fibers are created with a core of one polymer type and a shell of another [110,111]. By encapsulating honey within the fiber cores, its release could be delayed over time, with either diffusion or polymer degradation controlling its release rate. Templates created via this method should be subjected to rigorous mechanical testing to ensure that incorporating a honey core does not cause the fibers to weaken or become too brittle for their intended use. As of yet, there are no published studies using core-shell electrospinning with honey. However, this technology is likely a next step for the field. As there is no equivalent to core-shell electrospinning in the field of hydrogel and cryogel fabrication, other methods must be used to attenuate the honey release. Possible techniques include increasing polymer molecular weight and concentration and increasing the crosslinking density to reduce liquid diffusion through the templates—however, this will likely decrease the swelling ratio of the templates which may be undesirable. These effects will likely have to be balanced to achieve the optimal honey release rate, water vapor transmission rate, and absorbance of these hydrogels and cryogels. Additionally, due to the reported decrease in compressive modulus and strength of these constructs with honey incorporation, care will have to be taken to make sure that their mechanical properties are not compromised for their intended application [86,90,112].

More study is also needed on the effects of honey on immune cells such as neutrophils and monocytes/macrophages. While Tonks et al. showed that Manuka honey causes an increase in the output of TNF-α, IL-1, and IL-6 by monocytes over a 24-h period, it would be informative to ascertain the effect on levels of other inflammatory, anti-inflammatory, and angiogenic signals, such as IL-8, VEGF, IL-4, IL-1ra, MIP-1α, MIP-3α, etc. Additionally, similar testing of neutrophil cytokine output would be helpful, as their inflammatory and anti-inflammatory effects as the first responding immune cells in wounds are being assessed with increased importance [113,114,115]. Further testing of the effects of honey on neutrophil superoxide output, chemotaxis, and NF-κβ activation in the presence of different inflammatory and anti-inflammatory stimulators is needed to fully understand how honey affects the regulation of the wound environment by neutrophils. As noted earlier in this study, the current data suggests that high levels of honey (above 33% *w*/*v* for Manuka honey) are necessary to fight bacterial biofilms. However, the evidence also suggests that these high honey levels have the potential to cause significant cytotoxicity. Thus, in wounds with established biofilms, it is likely that a two-step treatment process will have to be implemented. First, a high concentration of honey can be applied topically to the wound to destroy the biofilm and eradicate the bacteria. After the infection has been eliminated, a tissue engineered template can be applied which releases lower levels of honey to reduce inflammation and aid in tissue regeneration, without causing cytotoxicity. In wounds without established biofilm-based infections, the first step may not be necessary, and the honey-eluting template may be directly applied to the area.

## 7. Conclusions

In vitro and in vivo evidence shows that honey, particularly Manuka honey, eliminates bacteria, resolves chronic inflammation, and promotes faster wound healing. Its potency against antibiotic-resistant bacteria, such as MRSA, makes it a particularly invaluable tool in an age where more strains of resistant bacteria are developing. As such, honey is a valuable addition to many tissue engineering templates in eliminating bacterial infection, aiding in inflammation resolution, and improving tissue integration with the template. Future research should focus on attenuating and prolonging the release of honey from the templates to avoid cytotoxicity and prolong the beneficial effects of the honey within the site.

## Figures and Tables

**Table 1:** Honeys inhibit bacterial growth. Zones of inhibition (diameter, in mm) of different concentrations of Ulmo and Manuka honey against various strains of MRSA. Standard deviations are shown in parentheses. “-” indicates no inhibition at that concentration. “*” indicates a clinical isolate. Reproduced with permission from Sherlock et al. *Complementary and Alternative Medicine*; published by BMC, 2010.

Concentration	50% *v*/*v*		25% *v*/*v*		12.5% *v*/*v*		6.3% *v*/*v*	
Isolates	Ulmo	Manuka	Ulmo	Manuka	Ulmo	Manuka	Ulmo	Manuka
MRSA ATCC 43300	30 (1.7)	24 (1.5)	26 (0.6)	19 (2.1)	18 (0.6)	13 (1.0)	10 (0.6)	-
MRSA 0791 *	34 (1.5)	23 (1.2)	29 (1.7)	17 (1.7)	22 (2.1)	-	14 (2.5)	-
MRSA 28965 *	24 (1.0)	17 (1.7)	19 (1.5)	15 (2.0)	-	-	-	-
MRSA 01322 *	28 (5.8)	22 (1.0)	23 (4.2)	18 (0.6)	17 (2.9)	-	11 (2.0)	-
MRSA 0745 *	23 (2.7)	20 (1.7)	19 (2.1)	13 (1.7)	11 (2.7)	-	-	-
*P. aeruginosa* ATCC 27853	14 (2.3)	16 (7.8)	11 (1.0)	14 (6.9)	-	-	-	-
*E. coli* ATCC 35218	14 (1.5)	15 (2.5)	11 (1.7)	12 (2.9)	-	-	-	-

**Table 2:** Summary of commercial wound-care products containing Manuka honey currently on the market.

Product Name	Company	Product Category
Medihoney^®^ Paste	Derma Sciences	Paste, topical application
Manuka Honey (Paste)	Manuka Health	Paste, topical application
Activon Tube	Advancis Medical	Paste, topical application
Medihoney^®^ Gel	Derma Sciences	Gel, topical application
ManukaApli	ManukaMed	Gel, topical application
Medihoney^®^ Alginate Dressing	Derma Sciences	Composite Hydrogel
Manuka Health Wound Dressing	Manuka Health	Composite Hydrogel
Algivon, Algivon Plus	Advancis Medical	Composite Hydrogel
Medihoney^®^ HCS Application	Derma Sciences	Honey-impregnated Dressing
Medihoney^®^ Honeycolloid Application	Derma Sciences	Honey-impregnated Dressing
Manukahd	ManukaMed	Honey-impregnated Dressing
ManukaMed MedSaf	ManukaMed	Honey-impregnated Dressing
Manukahd Lite	ManukaMed	Honey-impregnated Dressing
Manukahd Lite rope	ManukaMed	Honey-impregnated Dressing
Actilite	Advancis Medical	Honey-impregnated Dressing
Algivon Tulle	Advancis Medical	Honey-impregnated Dressing
